# Complete genome sequence of *Escherichia coli* phage vB_EcoP_EcoN5 isolated from Magdalena River, Atlántico, Colombia

**DOI:** 10.1128/mra.01269-25

**Published:** 2026-04-23

**Authors:** Dayan Lozano-Solano, Moisés Arquez-Mendoza, Solangie Quiñones-Vásquez, Camila Florez-Osorio, Nainy Quiroz, Cristian Solano, Antonio J. Acosta-Hoyos

**Affiliations:** 1 Centro de Investigaciones en Ciencias de la Vida-CICV, Universidad Simón Bolívar 125419, Barranquilla, Colombia; 2 Facultad de Ciencias Básicas y Biomédicas, Universidad Simón Bolívar 125419, Barranquilla, Colombia; 3 Estudiante de Maestría en Genética, Facultad de Ciencias Básicas y Biomédicas, Universidad Simón Bolívar 125419, Barranquilla, Colombia; 4 Estudiante de Microbiología, Facultad de Ciencias Básicas y Biomédicas, Universidad Simón Bolívar 125419, Barranquilla, Colombia; 5 Estudiante de Medicina, Facultad de Ciencias de la Salud, Universidad Simón Bolívar 125419, Barranquilla, Colombia; Portland State University, Portland, Oregon, USA

**Keywords:** bacteriophage assembly, *Escherichia coli*, antibiotic resistance, bacteriophage therapy

## Abstract

Antibiotic resistance represents a growing threat to public health, requiring alternative therapies. Here, we report the complete genome sequence and annotation of *Escherichia coli* bacteriophage vB_EcoP_EcoN5, isolated from river water in Barranquilla, Colombia. Genomic features suggest potential applications in future phage therapy strategies.

## ANNOUNCEMENT

Here, we describe the genome sequence and characterization of *Escherichia coli* phage vB_EcoP_EcoN5 (Eco N5)*.*

*EcoN5* was isolated using an *Escherichia coli* ATCC 25922 strain obtained from the Caribbean biobank of Universidad Simón Bolívar in Barranquilla, Colombia. We collected 10 mL of water from the Magdalena River in Barranquilla, Atlántico, Colombia (11.00636°N, −74.778415°W) on 18 September 2019, filtered through a 0.22 μm membrane, mixed with 10 mL of Lysogeny Broth (LB) and 100 µL of an overnight culture of *E. coli*, and incubated at 37°C with shaking at 180 RPM for 24 h. Phage plaques were propagated in LB medium supplemented with 0.05 mM CaCl_2_ using the double-layer overlay technique (1). The host range of EcoN5 was evaluated and shown to lyse an *E. coli* O157:H7 serotype by the double-layer overlay method (2).

Genomic DNA was extracted from 100 µL of filtered phage lysate obtained after propagation in a bacterial host culture, followed by clarification and filtration to remove residual cellular debris. DNA was purified using the PureLink Viral RNA/DNA Mini Kit (Thermo Fisher Scientific) according to the manufacturer’s instructions. Whole-genome sequencing followed a previously described workflow (3). Briefly, DNA was fragmented by ultrasonication to ~550 bp, and a library was prepared using the NEXTflex Rapid DNA Sequencing Kit. Fragments between 500 and 750 bp were selected using a BluePippin system (Sage Science), pooled with PhiX control DNA, and sequenced on an Illumina MiSeq platform using a v3 flow cell. DNA quantity and library integrity were assessed using a Qubit fluorometer and an Agilent 2100 Bioanalyzer.

The sequence generated 1,802,867 paired-end reads. Adapters and low-quality sequences (<Q30) were identified with FastQC (UseGalaxy.eu) and removed with Trimmomatic, along with reads <50 bp. Filtered reads were assembled *de novo* using SPAdes version 3.11.1 (4). The assembly yielded a 76,083 bp contig with the highest k-mer coverage (14.2×), consistent with the complete phage genome. Annotation with RAST-Tk version 1.073 (5) identified coding DNA sequences (CDSs) and predicted putative functions; proteins lacking annotation were further analyzed with BLASTp (6) and HHpred (7).

**Fig 1:**
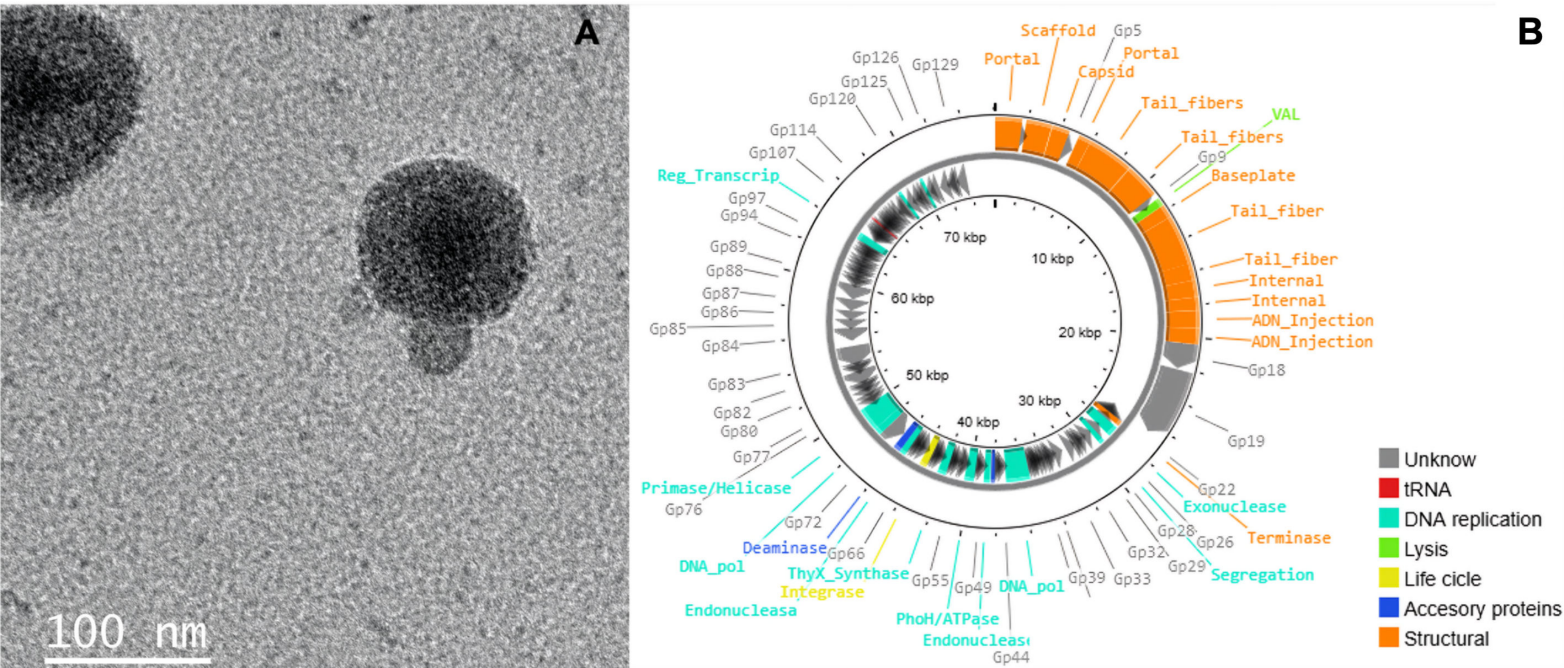
(**A**) Virion morphology and (**B**) genome modular structure of bacteriophage vB_EcoP_EcoN5. The virion morphology was determined through electron microscopy. A high-titer lysate was applied to formvar-coated grids, negatively stained with 2% uranyl acetate at an accelerating voltage of 200 kV, and 195,000× magnification was used. The images were captured with a Zeiss EM-109 transmission electron microscope (Carl Zeiss AG), corresponding to a Podovirus. (**B**) The genomic map was generated using Proksee (13) based on the EcoN5 annotated genome.

BLAST analysis showed high sequence similarity between EcoN5 and phages Paul (98%) (MN045231) (8), NJ01 (95.5%) (NC_018835.1) (9), and phiEco32 (98.8%) (NC_010324.1) (10). EcoN5 displays a podovirus morphotype (Fig. 1A) and has a 76,083 bp genome with a GC content of 42.09%. A total of 128 CDSs were predicted, of which 40 had putative functions. The genome is organized into functional modules based on RAST-Tk subsystem categorization and manual curation with BLASTp and HHpred (Fig. 1B). These include modules for DNA regulation and replication (23 CDSs), structural and assembly proteins (10 CDSs), the lysis module (3 CDSs), and life cycle regulation (3 CDSs), including an integrase indicative of lysogeny. The genome also encodes a rare tRNA with the UCU anticodon for arginine (11). Terminal redundancy and circular permutation consistent with headful packaging were identified using PhageTerm2 (12). Default parameters were used unless otherwise noted.

## Data Availability

The genome sequence of phage EcoN5 was deposited in GenBank under the accession number MN715356. The raw sequence reads have been submitted to the NCBI SRA under accession number SRR33084599.
